# Tumor RNA-transfected dendritic cells and combined therapy with low dose 5-FU induce regression of murine colon cancer

**DOI:** 10.1186/1753-6561-7-S2-P45

**Published:** 2013-04-04

**Authors:** Marcela R de Camargo, Carolina M Gorgulho, Cecília P Rodrigues, Juliana CL Frederico, Fabiana A Zambuzi, Victoria E Galvão, Marcimara Penitenti, Ramon Kaneno

**Affiliations:** 1 Department of Microbiology and Immunology, Institute of Biosciences, UNESP, Botucatu, SP, Brazil; 2 Department of Pathology, School of Medicine, UNESP, Botucatu, SP, Brazil; 3 Fundação Amaral Carvalho, Jaú - SP, Brazil

## Background

We have recently observed that treatment of colon tumor cells with low concentration of paclitaxel increased the expression of several genes associated with antigen-presenting machinery. Since 5-fluoruracil (5-FU) is the main antineoplastic agent for colon cancer, in this study we aimed to evaluate: a) whether transfection of dendritic cells (DC) with drug-treated tumor cells RNA, enhances the effectiveness of DC-based vaccine; b) if the modulatory effects of vaccine can be observed *in vivo*, and c) if the combination of DC with low dose chemotherapy schedule improves the antitumor responsiveness.

## Materials and methods

Murine colon cancer cells (MC-38) were treated with the minimum effective concentration (MEC) of 5-FU and their RNA was used to transfect DC. Then, C57/Bl-6 tumor-bearing mice were treated with DC vaccine. Another group of animals received low doses of chemotherapy schedule and DC vaccine.

## Results

Results of 2 independent assays have shown that vaccination with RNA-transfected DC delayed the tumor growth, increased the percentage of CD86+ (35%) CD40+ (63%) and MHC class II+ (47%) and significantly increased the *in vitro* production of IFN-γ and decreased IL-10 by spleen cells co-culture. In addition we observed that the combination of DC vaccine with low dose 5-FU chemotherapy induced complete tumor regression in 75% of the treated animals.

## Conclusion

Taken together our results indicate that low dose 5-FU plus DC vaccine can enhance the antitumor response and lead to complete tumor regression.

## Finacial support

FAPESP 2009/18331-8; 2010/06013-9.

